# RAD21 Cooperates with Pluripotency Transcription Factors in the Maintenance of Embryonic Stem Cell Identity

**DOI:** 10.1371/journal.pone.0019470

**Published:** 2011-05-12

**Authors:** Anja Nitzsche, Maciej Paszkowski-Rogacz, Filomena Matarese, Eva M. Janssen-Megens, Nina C. Hubner, Herbert Schulz, Ingrid de Vries, Li Ding, Norbert Huebner, Matthias Mann, Hendrik G. Stunnenberg, Frank Buchholz

**Affiliations:** 1 Max Planck Institute of Molecular Cell Biology and Genetics, Dresden, Germany; 2 Department of Molecular Biology, Nijmegen Centre of Molecular Life Sciences, Radboud University, Nijmegen, Netherlands; 3 Max-Delbrueck Center of Molecular Medicine, Berlin, Germany; 4 Max Planck Institute of Biochemistry, Martinsried, Germany; Florida State University, United States of America

## Abstract

For self-renewal, embryonic stem cells (ESCs) require the expression of specific transcription factors accompanied by a particular chromosome organization to maintain a balance between pluripotency and the capacity for rapid differentiation. However, how transcriptional regulation is linked to chromosome organization in ESCs is not well understood. Here we show that the cohesin component RAD21 exhibits a functional role in maintaining ESC identity through association with the pluripotency transcriptional network. ChIP-seq analyses of RAD21 reveal an ESC specific cohesin binding pattern that is characterized by CTCF independent co-localization of cohesin with pluripotency related transcription factors Oct4, Nanog, Sox2, Esrrb and Klf4. Upon ESC differentiation, most of these binding sites disappear and instead new CTCF independent RAD21 binding sites emerge, which are enriched for binding sites of transcription factors implicated in early differentiation. Furthermore, knock-down of RAD21 causes expression changes that are similar to expression changes after Nanog depletion, demonstrating the functional relevance of the RAD21 - pluripotency transcriptional network association. Finally, we show that Nanog physically interacts with the cohesin or cohesin interacting proteins STAG1 and WAPL further substantiating this association. Based on these findings we propose that a dynamic placement of cohesin by pluripotency transcription factors contributes to a chromosome organization supporting the ESC expression program.

## Introduction

The cohesin complex, consisting of four core subunits (SMC1a, SMC3, RAD21 and STAG (STAG1 or STAG2)), is important for a variety of biological processes including chromosome segregation, DNA-damage repair and chromosome morphology [1], [2], [3], [4], [5], [6]. A model of a ring-like structure suggests that cohesin can encircle DNA, thereby physically connecting different DNA strands for these diverse biological processes. Cohesin functions are supported by accessory proteins such as the cohesin loading factor NIPBL [7], [8] as well as the cohesin maintenance proteins WAPL [9], [10] and PDS5 [11], [12].

Recent studies have extended the canonical functions to a role of cohesin in gene regulation [13], [14], [15], [16], [17], [18], [19], [20]. For instance, experiments have revealed that cohesin is loaded onto chromatin long before sister-chromatid cohesion is established [14]. Furthermore, cohesin is present in postmitotic cells and only a small amount is actually needed for mitosis [21], [22]. Association of cohesin with human developmental disorders occurring relatively late in development also proposed a functional role in gene expression regulation, because mutations that only affect sister-chromatid cohesion would predict to cause lethality early in development. However, disorders displaying mutations in the cohesin network termed cohesinopathies do not show severe defects in sister chromatid cohesion [14], [23], [24], [25]. Finally, the prominent co-localization with the chromatin boundary factor CTCF suggests a role of cohesin in chromatin mediated gene regulation [14], [24], [26], [27], [28], [29], [30], [31]. However, how cohesin contributes to the execution of specific gene expression programs is not well understood.

Interestingly, we and others recently identified cohesin subunits in RNAi ESC screens as factors that are required to maintain Oct4 expression [32], [33]. ESCs present an excellent cellular system to investigate a potential role of cohesin in gene regulation because the transcriptional repertoire that maintains ESC identity has been studied extensively and conditions for an exit from the self-renewal program are well defined.

## Results

### An organizational principle of RAD21 at CTCF binding sites

To gain insights into a potential role of cohesin in ESC biology, we performed a global DNA-binding survey of RAD21, a core component of the cohesin complex. We used a RAD21 BAC-GFP tagged ESC line and performed chromatin immunoprecipitation followed by massive parallel DNA sequencing (ChIP-seq) employing a high-affinity GFP-antibody. Applicability and specificity of the BAC tagging approach for generic assays has been demonstrated previously [34], [35] and has been specifically validated here for RAD21 (Fig. S1). For analysis of the ChIP-seq data set, we first subtracted the IgG ChIP-seq data from the Rad21 ChIP-seq data and employed the MACS algorithm (1.4.0beta version) to call binding peaks. With a cut-off *p*-value of 10e-5, we identified 15311 specific RAD21 binding sites (Table S1, Fig. S2) unmasking a characteristic binding pattern of RAD21 in ESCs.

**Figure 1:**
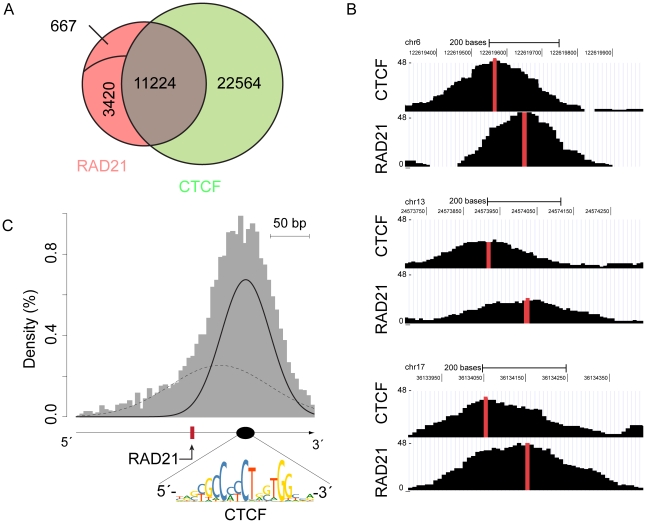
Organizational principle of RAD21 placement near CTCF binding sites in ESCs. (**A**): Venn diagram showing the overlap of RAD21 with CTCF binding sites (p<10^−5^). Non-overlapping RAD21 binding sites are separated into CTCF binding independent (3420, named CIB) and CTCF binding and motif independent sites (667, named MCIB). (**B**): Examples of ChIP-seq binding peaks indicating a shift of RAD21 and CTCF loci. Vertical red bars indicate the CTCF binding peak summits. Data show absolute number of reads at the y-axis and chromosome position at the x-axis. (**C**): Histogram of distances between RAD21 binding sites and CTCF strand-specific motifs identified RAD21 to be located at CTCF binding sites with upstream directionality. Black lines are the components of the Gaussian mixture modeling distribution of distances. The solid line indicates the most dominant distribution and the dashed line indicates a second component of the mixture model. The red bar indicates the RAD21 binding site and the black ellipse indicates position of CTCF binding motif depicted below.

Consistent with genome-wide binding studies for other cohesin subunits [24], [28], inspection of the bound sequences did not reveal a RAD21 specific consensus sequence, indicating that the cohesin complex is not a sequence specific DNA binding complex. Instead, we identified the CTCF binding motif in 93% of all RAD21 binding sites. Cohesin has been shown to co-localize with the insulator protein CTCF, where it most likely contributes to chromosome organization via DNA loop formation between distant genomic regions [24], [30], [36]. To test whether this co-localization is indeed present in ESC, we also conducted ChIP-seq analysis with a validated CTCF BAC-GFP tagged cell line (Fig. S1). Using the same parameters for peak detection, we identified 33788 CTCF binding sites in ESCs. We compared the RAD21 binding pattern to the CTCF binding sites in ESC and observed a remarkable overlap of the binding patterns of these two factors, with 73% of the RAD21 binding sites co-localizing with CTCF binding sites (Fig. 1A).

Close inspection of the RAD21 and CTCF peaks revealed that RAD21 typically does not exactly overlap with the CTCF binding position. Instead, RAD21 peaks were slightly shifted in respect to the CTCF summits (Fig. 1B). For a comprehensive analysis of this observation, we computationally determined the binding sites integrating all RAD21 peaks and calculated the distance to the closest CTCF binding motif in a strand specific manner. This analysis validated our initial observation and uncovered a directional placement of RAD21 5′ of the CTCF motif (Fig. 1C). Thus, our study reveals an hitherto unknown organizational principle of RAD21 binding close to CTCF sites *in vivo*.

### CTCF-independent RAD21 binding sites preferentially co-localize with key pluripotency related transcription factors

**Figure 2:**
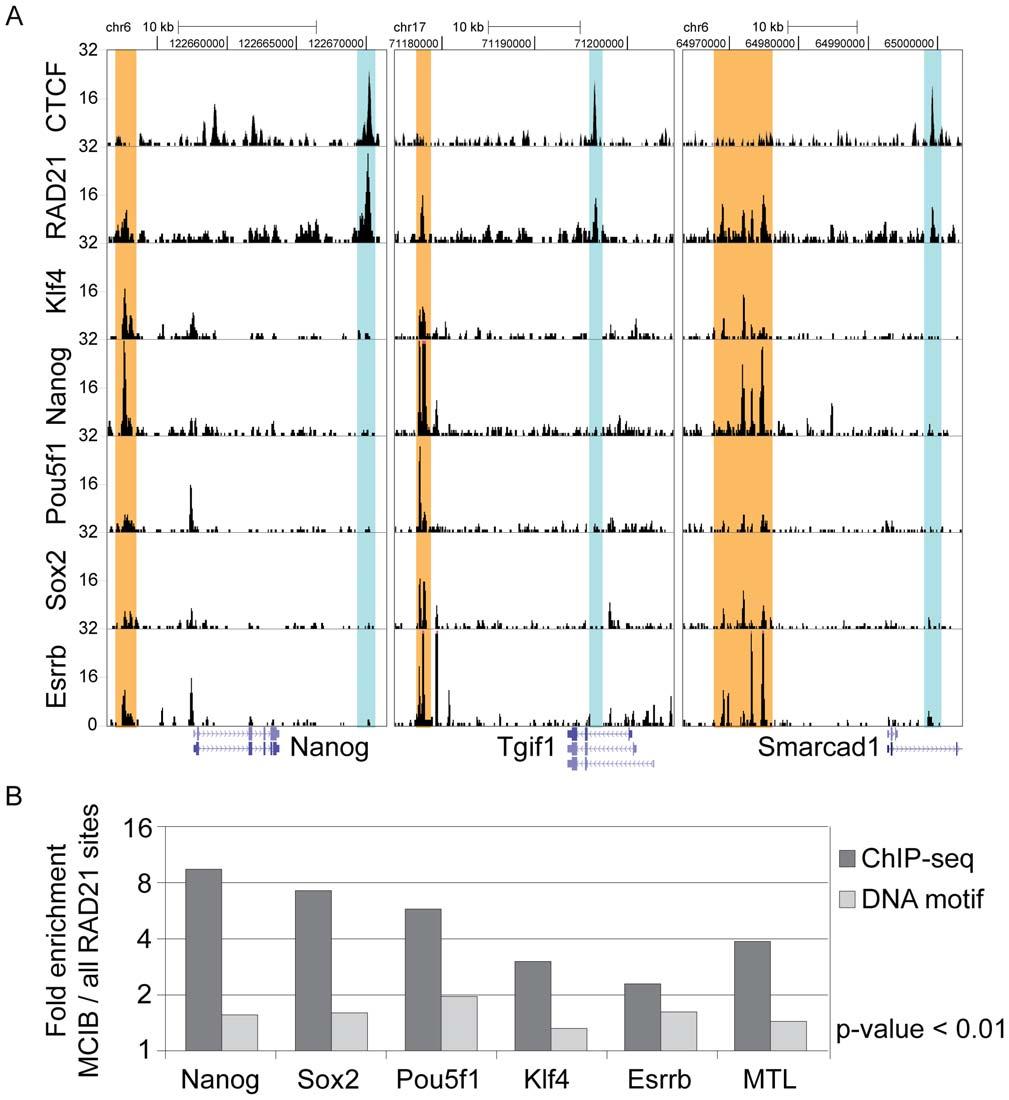
MCIB RAD21 binding sites co-localize with key pluripotency related transcription factors. (**A**) Examples of ChIP-seq results showing co-localization of MCIB RAD21 binding sites with key ESC specific transcription factors. ChIP-seq data were obtained from previous studies [37] for the indicated loci in ESCs. MCIB RAD21 sites are highlighted in red and CTCF-overlapping sites are highlighted in blue. (**B**) Enrichment analysis of transcription factors in the 150 bp vicinity of MCIB RAD21 binding peak summits over all RAD21 binding peak summits shows overrepresentation of both DNA binding motifs and real binding of ESC specific transcription factors at MCIB RAD21 sites. MTL (Multiple Transcription Factor loci) are binding sites with at least two pluripotency transcription factors that co-localize with RAD21. Black bars show enrichment of binding events identified by ChIP-seq [37] and grey bars show enrichment of DNA binding motifs.

Despite the high overlap of RAD21 and CTCF binding, we also identified 4087 (26.7%) RAD21 binding sites that did not co-localize with CTCF in ESCs (Fig. 1A and Table S2). This result is consistent with previous studies in somatic cells, where non-CTCF bound sites for different cohesin components were reported [14], [28], [30]. Closer inspection of these binding sites revealed that 3420 (83.7%) of the non-CTCF bound binding sites contained the CTCF motif, indicating that they are either not bound by CTCF in ESCs, even though the binding motif is present or that these sites were not detected during ChIP-sequencing. To generate a list of unambiguous CTCF negative RAD21 binding sites we removed these sites, leaving 667 reassessed CTCF independent RAD21 binding sites, which we define as motif and CTCF-independent RAD21 binding sites (MCIB). To investigate whether other binding sites could be detected in this MCIB subset, we performed a motif search analysis in the vicinity (150bp) of MCIB RAD21 peak summits (Table S2). Strikingly, this analysis showed an enrichment for DNA motifs that are known binding sites of key pluripotency transcription factors (Fig. 2B). In particular, bona-fide binding motifs for Oct4 (encoded by Pou5f1), Nanog, Sox2, Klf4, and Esrrb were enriched at MCIB over all RAD21 binding sites. To investigate whether RAD21 does indeed co-localize with pluripotency transcription factors at these sites *in vivo*, we compared our data with published ChIP-seq data [37] of pluripotency transcription factors in ESCs. Remarkably, a significant overlap of MCIB RAD21 binding sites and the ESC transcription factors with a clear enrichment of ESC transcription factors at these particular MCIB sites over all RAD21 sites was observed (Fig. 2, Table S3). Moreover, many of the MCIB RAD21 sites co-localized with Multiple Pluripotency Transcription Factor-Binding loci (MTL), from which around 40% were reported to be located in ESC intergenic regions [37] (Fig. 2A, Table S3). Interestingly, the MCIB RAD21 binding sites did not show a characteristic directional binding to the pluripotency transcription factor motifs as seen for CTCF co-bound sites.

### RAD21 and other cohesin subunits are required to maintain ESC identity

**Figure 3:**
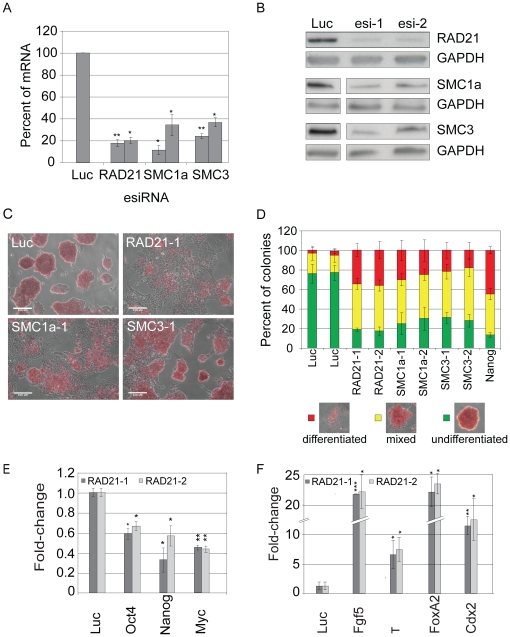
Depletion of RAD21 and other cohesin subunits leads to differentiation of ESCs. (**A**): qPCR analysis of esiRNA knock-down efficiency for indicated genes (48 h RNAi, n = 3, error bars denote s.d. and *, ** indicate p<0.05 and 0.01 respectively). Transfection of a non-targeting esiRNA (Luc) was used as a control. Numbers adjacent to the gene names indicate independent, non-overlapping esiRNAs transfected. (**B**): Western Blot analysis of esiRNA knock-down efficiency for indicated genes (48 h RNAi; esi-1 and esi-2 indicate independent, non-overlapping esiRNAs transfected). Transfection of a non-targeting esiRNA (Luc) was used as a control. GAPDH served as a protein loading control. (**C**): Alkaline phosphatase staining of ESCs, which had been transfected with esiRNAs targeting RAD21, SMC1a and SMC3 (72 h post RNAi) showed a strong differentiation phenotype compared to a control (Luc). Nanog depletion served as a positive control for ESC differentiation. Scale bars correspond to 100 µm. (**D**): Quantification of alkaline phosphatase staining (n = 3) separated into undifferentiated (green), mixed (yellow) and differentiated colonies (red). (**E+F**): qPCR validation of expression changes of (**E**) stem cell maintenance genes and (**F**) lineage marker genes upon knock-down of RAD21 (two independent esiRNAs) and Luc control (48 h RNAi, n = 3, error bars denote s.d. and *, **, *** indicate p<0.05, 0.01 and 0.001 respectively).

The co-localization of RAD21 with pluripotency transcription factors prompted us to further investigate the functional role of RAD21 and other cohesion subunits in ESC maintenance. Because of its essential role during cell division, complete removal of cohesin causes cell death [38], [39], [40]. However, knock-down of cohesin complex components by RNAi has been used to investigate non-essential functions of the complex for instance in MCF-7 cells [28]. These experiments revealed that cells could divide and survive with reduced levels of cohesin proteins [22]. As in MCF-7 cells, transfection of ESCs with esiRNAs targeting RAD21, SMC1a or SMC3 resulted in significant depletion both at mRNA and protein level (Fig. 3A,B) without causing pronounced cell death (data not shown). Importantly, ESC colonies lost their typical compact morphology 72 h post transfection with many flat and extended cells appearing at the periphery of colonies (Fig. S4), suggesting loss of pluripotency. Alkaline phosphatase staining showed a strong reduction of AP-positive cells for knock-down of all three subunits, verifying the loss of pluripotency and subsequent differentiation upon cohesin depletion (Fig. 3 C,D, S5A). The loss of AP expression upon RAD21 depletion was accompanied by a decrease in expression of pluripotency markers, including Oct4, Nanog and Myc (Fig. 3E). Conversely, the expression of lineage markers characteristic for all three germ-layers (ectoderm: Fgf5, mesoderm: brachyury, endoderm: FoxA2) and trophectoderm (Cdx2) were strongly upregulated, signifying differentiation of the cells (Fig. 3F).

To test whether the expression change of pluripotency and early developmental genes in RAD21 depleted cells is also valid for other cohesin subunits, we repeated the expression analysis with SMC1a and SMC3 depleted ESCs and detected similar expression changes of pluripotency and lineage markers (Fig. S5 B,C). Although the magnitude of expression changes for some markers varied, possibly due to experimental variations, differences in knockdown efficiency or divergence in protein half-life, the overall concordance of the results were striking, indicating that the observed phenomena can be attributed to the cohesin complex rather than RAD21 itself. In summary, physiological expression of cohesin is required to maintain ESC identity.

### Expression changes after RAD21 depletion resemble Nanog knock-down

**Figure 4:**
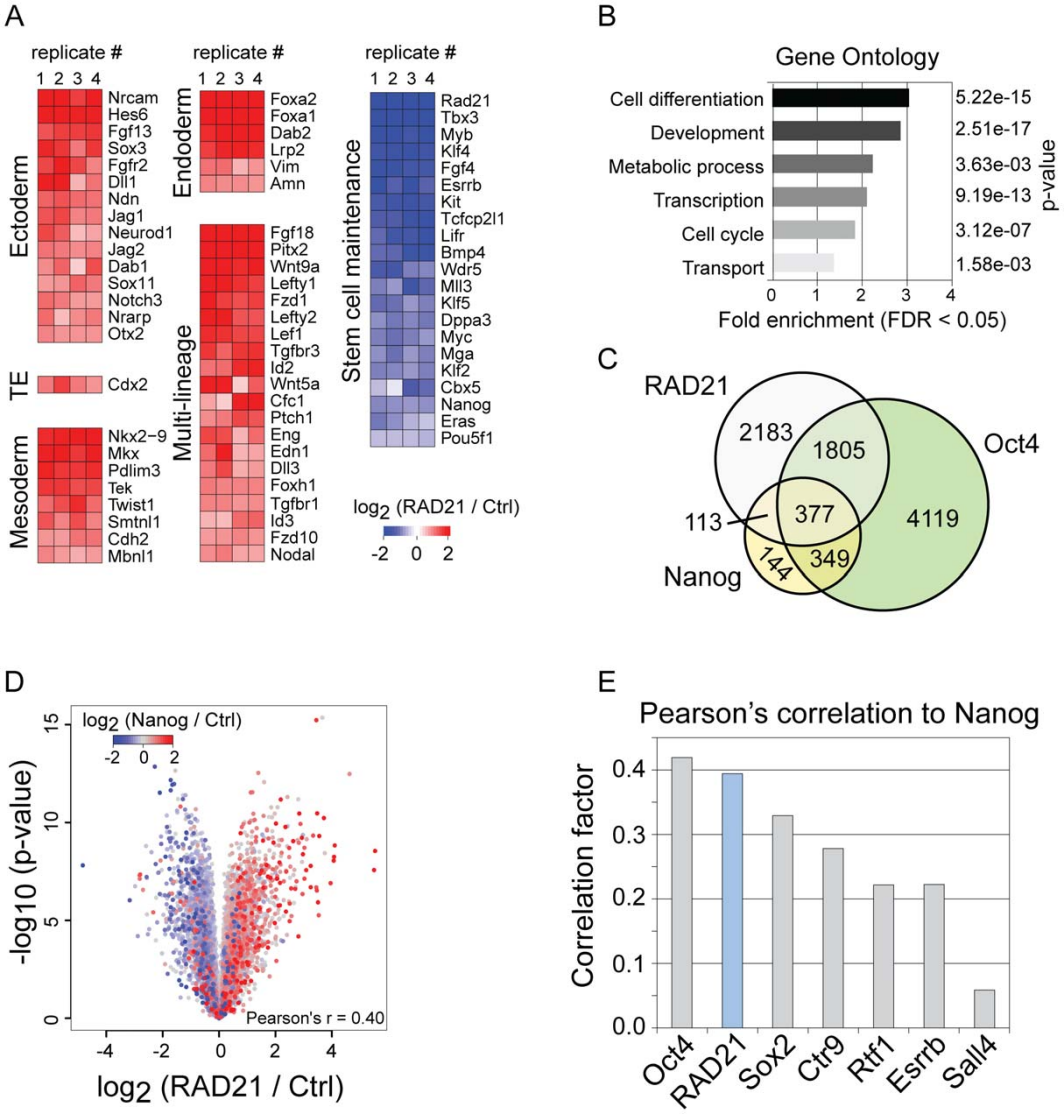
Gene expression profile upon RAD21 depletion exhibits close analogy to Nanog depletion. (**A**): Heatmap showing expression changes of selected developmental and stem cell maintenance genes in four biological replicates (48 h RNAi). (**B**): Gene Ontology (GO)-term analysis determined ‘Cell differentiation’ and ‘Development’ as processes to be most overrepresented. (**C**): Venn diagram showing the overlap of RAD21 with pluripotency genes Oct4 and Nanog expression profiles after their depletion. (**D**): Volcano plot demonstrating the remarkable overlap of RAD21 and Nanog expression profiles upon their depletion. Each dot represents a probe from the RAD21 microarray (averaged over four replicates), blue dots represent downregulated genes and red dots represent upregulated genes. (**E**): Bar chart of Pearson's correlation factors between expression profiles of Nanog and factors essential for stem cell identity after their depletion. Note that the correlation factor for RAD21 is similar to the correlation factors for Oct4 and Sox2.

To obtain a global view of expression changes, we transfected ESCs with RAD21 esiRNAs and measured expression changes after 48 hours using microarrays. Strong changes of the transcriptome were evident (Table S4), supporting a role of RAD21 in ESC gene regulation. More importantly, many known stem cell maintenance genes, including Tbx3, Esrrb, Klf4, Fgf4, Nanog and Oct4 were downregulated (Fig. 4A, S6). In contrast, a large number of developmental genes from all three germ layers and the trophectoderm lineage were upregulated (Fig. 4A, S6). Notably, many genes associated with known critical ESC maintenance signaling pathways, including the TGF-β, Wnt and Notch signaling pathways were also affected (data not shown). Gene Ontology enrichment analysis showed that genes associated with cell differentiation and development were strongly enriched (p>5×10^15^) (Fig. 4B), manifesting the exit of the pluripotency expression program and induction of differentiation programs upon RAD21 knock-down.

To place the observed expression changes into context of other factors, we compared the RAD21 expression profile to published data [41], [42]. Remarkably, the expression profile after RAD21 knock-down was similar to expression changes reported for the depletion of pluripotency transcription factors (Fig. 4C). Thus, RAD21 knock-down mirrors expression changes observed after depletion of pluripotency transcriptional regulators. In particular, the depletion of RAD21 closely resembled the expression profile after Nanog knock-down with a Pearson correlation coefficient of 0.4 (Fig. 4D,E). Notably, this correlation coefficient matched or exceeded a comparison between Nanog and other pluripotency factors, indicating a particularly tight link between Nanog and RAD21 depletion (Fig. 4E). This finding, together with the observed phenotypical consequences upon knock-down and the observed co-localization at MCIB RAD21 sites suggests that RAD21 is linked to the maintenance of ESCs through an association with pluripotency transcription factors.

### Co-localization of RAD21 with pluripotency related transcription factors at CTCF-independent sites is specific for ESCs

**Figure 5:**
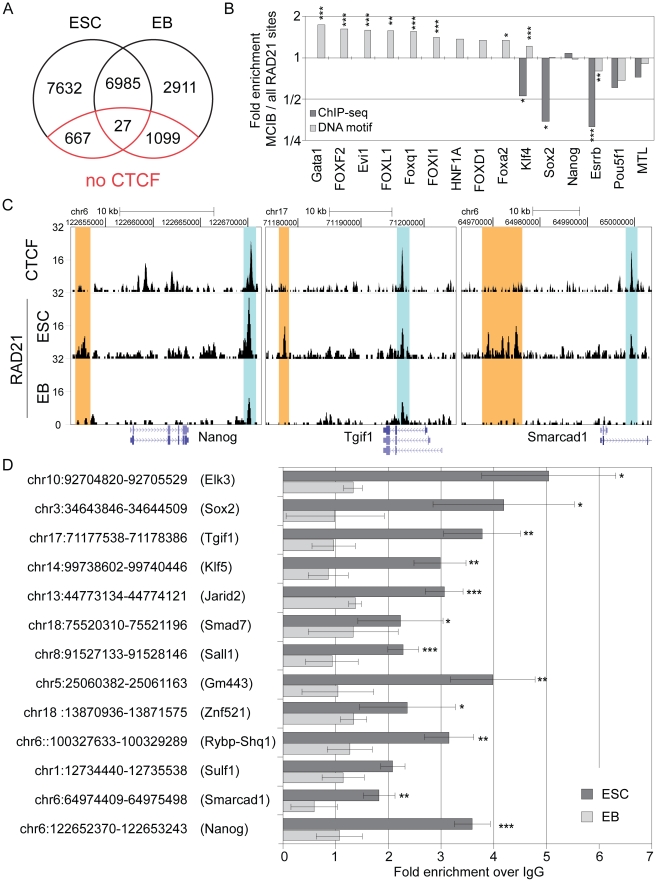
MCIB RAD21 binding sites are predominantly specific for ESC and disappear in EBs. (**A**): Venn diagram displaying the number of RAD21 binding sites specific for ESCs and EBs as well as the number of overlapping binding sites. The red circle separates the MCIB binding sites. (**B**): Enrichment analysis of transcription factors at MCIB over all RAD21 binding sites in EBs indicate upregulation of developmental- and downregulation of ESC specific transcription factors. Black bars show enrichment of binding events identified by ChIP-seq and grey bars show enrichment of binding motifs (*, **, *** indicate Fisher's exact test p<0.05, 0.01 and 0.001, respectively). (**C**): Examples of ChIP-sequencing peaks in ESCs and EBs showing the disappearance of MCIB RAD21 binding sites that co-localize with ESC transcription factors. MCIB RAD21 sites are highlighted in red and CTCF-overlapping sites are highlighted in blue. (**D**): Validation of selected loci by conventional ChIP followed by quantitative real-time PCR (n = 3, error bars denote s.d. and *, **, *** indicate p<0.05, 0.01 and 0.001, respectively). Black bars show fold enrichment in ESCs and grey bars show enrichment in EBs. Labels indicate the chromosome positions of the target loci and the closest gene indicated in brackets.

Functional relevance of RAD21 co-localizing with pluripotency maintaining transcription factors for ESC identity would suggest that these binding sites are ESC specific and that RAD21 would not be found there when cells exit the pluripotency program. To investigate this assumption, we differentiated LAP-tagged RAD21 ESCs into embryoid bodies (EBs) and performed ChIP-seq using the same parameters as employed previously for ESCs. With this analysis, we detected 11022 RAD21 binding sites in EBs. Interestingly, around half of the 15311 binding sites detected in ESCs, namely 7012 (45.8%), were also detected in EBs. This result suggests that many of the RAD21 binding sites are maintained upon differentiation (Fig. 5A). In support for this observation, a lot of the unaltered RAD21 binding sites in ESCs are also conserved in B- and T- cells [24] (data not shown). In sharp contrast, the majority of the 667 MCIB RAD21 sites including the MTL co-localizing sites in ESCs had disappeared in EBs, indicating that the co-localization of RAD21 with pluripotency transcription factors is specific for ESCs (Fig. 5A–D). At the same time DNA binding motifs of transcription factors related to early development were now enriched at the MCIB RAD21 binding sites (Fig. 5B), suggesting that these transcription factors now cooperate with cohesin to influence gene expression. As for the MCIB RAD21 binding sites in ESCs, we did not observe directional binding to transcription factor motifs (Fig. S3) as seen for CTCF co-bound sites.

### Nanog interacts with cohesin proteins STAG1 and WAPL

**Figure 6:**
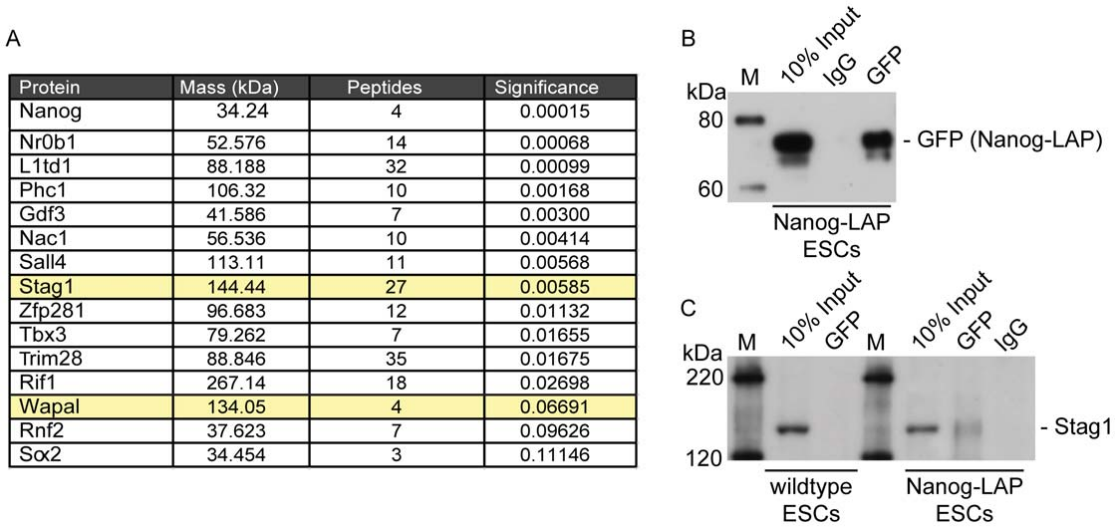
Nanog interacts with STAG1 and the cohesin associated protein WAPL. (**A**): List of selected proteins identified by mass spectrometry of Nanog BAC-GFP tagged ESCs. Proteins with their masses, identified number of peptides and significances (student's t-test) are shown. The cohesin protein STAG1 and cofactor WAPL are highlighted in yellow. (**B**): Western blot analysis after co-immunoprecipitation of GFP in Nanog BAC-GFP tagged ESC detected the bait (Nanog-LAP) using a GFP-antibody. Precipitation with an IgG antibody served as a control. (M = protein marker, kDa) (**C**): Western blot analysis after co-immunoprecipitation of GFP in Nanog BAC-GFP tagged ESCs and wildtype ESCs revealed a specific interaction with STAG1 in the GFP-IP. No interaction was detected in wildtype ESCs (M = protein marker, kDa).

Because cohesin by itself does not seem to bind DNA sequence specifically, other mechanisms to place it at specific regions in the genome must exist. For RAD21 binding at CTCF sites, CTCF is suggested to localize cohesin via protein-protein interaction with STAG (SCC3) [30]. In order to gain insight into the mechanism of how cohesin is recruited to the ESC transcription factor binding sites, we performed a proteomic study on Nanog, the transcription factor with the highest enrichment score and the most similar expression pattern changes upon RAD21 depletion. Immunoprecipitation of LAP-tagged Nanog protein in Nanog-LAP- and control wildtype ESCs identified well known interaction partners of Nanog including Nr0b1, Zfp281, Sall4, Nac1, Gdf3, Sox2 and Rnf2 and thus validated functionality of the Nanog-LAP ESC line (Table S5). Interestingly, we also detected the core cohesin protein STAG1 and the cohesin associated protein WAPL as interaction partners of Nanog (Fig. 6A). To validate this result, we tested the interaction of STAG1 with Nanog by co-immunoprecipitation of GFP versus an IgG control in Nanog-LAP ESCs as well as in wildtype ESCs and confirmed the specific interaction in Nanog-LAP cells (Fig. 6B,C). Collectively, these data indicate that Nanog may facilitate the placement of cohesin at its binding sites through an interaction with specific cohesin proteins and together with similar phenotypical consequences upon knock-down and co-localizing DNA-regions, suggests cooperativity between the non-specific DNA-sequence bound (chromatin organizer) cohesin and the specific DNA-sequence bound pluripotency transcription factors.

### Comparative DNA binding analysis of cohesin sub-units

**Figure 7:**
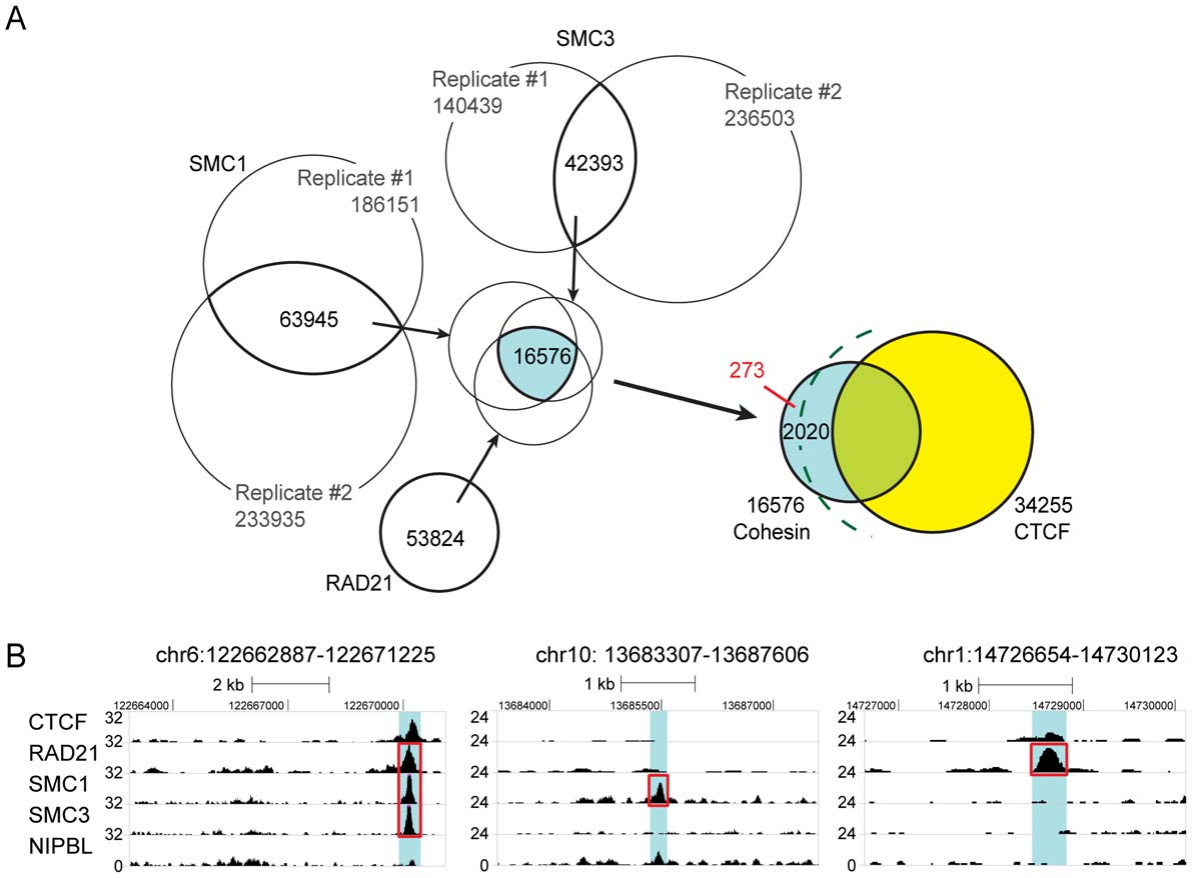
Integrated analysis of RAD21, SMC1 and SMC3 binding sites in ESCs. (**A**): Common binding sites of RAD21, SMC1 and SMC3 [43] and the overlap with common CTCF sites are shown. The number of binding sites are depicted for each experiment and the overlap of the data is presented in the intersection. The green dashed line separates cohesin sites that are independent of CTCF from sites that are independent of CTCF binding and do not contain the CTCF motif sequence (MCIB, shown in red). (**B**): Examples of ChIP-sequencing peaks in ESCs showing co-localization of all sequenced cohesin subunits and localization of individual subunits only. Areas of interest are marked in blue and called cohesin subunit binding sites are boxed in red. Chromosomal locations of the peaks are indicated above the graphs. Note the absence of peaks for RAD21 and SMC3 in the second, and the absence of peaks for SMC1 and SMC3 in the third example, respectively.

Recently, the binding pattern of the cohesin subunits SMC1a and SMC3 were described in ESCs [43]. We re-analyzed these data sets (two replicates each, Fig. S7) and integrated our RAD21 data using the MACS algorithm (p<10^−2^) to calculate the number of common cohesin binding sites (Fig. 7A), creating a list of high confidence common cohesin binding sites in ESCs (Table S7). We also integrated the different CTCF data sets to determine the number of common cohesin binding sites that are independent of CTCF. This analysis identified 16576 common cohesin binding sites, from which 2020 (12%) sites were independent of CTCF binding but still exhibit the CTCF motif sequence and 273 (1.7%) sites that are independent of CTCF binding and do not contain the CTCF motif sequence (MCIB). This result indicates that the number of cohesin binding sites that are independent of CTCF is lower then the number of sites where these proteins co-localize. This number becomes substantially smaller when also the sites that still contain the CTCF motif sequence are considered in this analysis.

Interestingly, the comparative analysis revealed binding sites of individual subunits that are not shared with other cohesin subunits (Fig. 7B). This observation raises the possibility that, apart from a major concordance of core cohesin binding sites, individual cohesin subunits may bind to DNA in a unique manner. However, it is also possible that this observation is a result of the different ESCs and antibodies employed in the different studies or may also reflect experimental variability in ChIP-seq experiments.

## Discussion

Cooperativity between mammalian transcription factors that bind sequence specific DNA elements and factors that mediate chromosome conformation have long been proposed, but molecular details on how they work together have been sparse. The recent implication of cohesin in regulating chromosome conformation [20], [27], [36], [44] together with its detection in ESC identity RNAi screens [32], [33] prompted us to investigate the role of RAD21 in ESCs in more detail. Our genome-wide RAD21 binding survey in ESCs and EBs revealed three modes of RAD21 binding, the conserved and the dynamic CTCF dependent and the CTCF independent binding mode.

The majority of sites that are co-occupied by CTCF and RAD21 appear to be conserved in ESCs and in different cell types, suggesting that they may provide a framework supporting a general chromosome architecture. Our observation that RAD21 exhibits a characteristic shifted directionality with respect to CTCF, which we did not observe for pluripotency transcription factor co-bound sites, might indicate a different functionality of RAD21 at the CTCF dependent compared to the independent sites or a different mode of physical interaction with the particular transcription factors. Physical interaction of cohesin with CTCF has recently been reported [30], suggesting a direct role of CTCF in recruiting RAD21 5′ of its binding site, although factors that mediate the placement cannot be excluded. In this context, a methylation dependent mechanism may account for recruiting cohesin because CTCF binds DNA in a methylation specific manner [16]. It will be interesting to further investigate how this placement is achieved and what relevance it has with respect to transcriptional insulation and chromosome architecture.

The comparative RAD21 binding analysis in ESCs versus EBs also uncovered changes in the CTCF dependent RAD21 binding sites, revealing the dynamic CTCF dependent RAD21 binding mode mentioned above. The fact that these sites change upon differentiation indicates that they may be important during development. Since the epigenetic chromatin state changes with differentiation [45], the previously described DNA methylation dependency of CTCF binding [16] may account for the change of dynamic CTCF dependent binding of RAD21 in EBs. To further investigate this aspect, it will be interesting to compare the epigenetic changes with CTCF and cohesin binding sites during ESC differentiation in the future.

Probably the most intriguing finding was the identification of the motif and binding CTCF independent (MCIB) RAD21 binding sites that exhibited enrichment for pluripotency related transcription factors binding sites. The fact that these sites disappear in EBs and new binding sites emerge, which are enriched for early developmental transcription factors suggests that these sites are functionally relevant for the execution of developmental expression programs. The expression changes observed after RAD21 depletion mirroring knockdown of Nanog further support this notion.

Notably, the number of CTCF independent RAD21 binding sites in ESCs is considerably lower than reported for other cohesin subunits, namely SMC1a and SMC3 [43]. However, this is largely due to the different analysis methods employed and re-analysis of the data with our parameters also resulted in a much lower percentage of CTCF independent SMC1 and SMC3 binding sites including MCIB sites. Moreover, our comparative analysis revealed binding sites of individual subunits that are not shared with other cohesin subunits, suggesting that individual cohesin subunits are found at certain DNA sites. Whether this observation indeed reflects independent subunit binding or instead is a result of the different ESCs and antibodies employed or experimental variability in ChIP-seq experiments needs to be determined in the future. Nonetheless, independent of the analysis method employed, the cumulative data suggests that RAD21 together with the other cohesin proteins maintains ESC identity by connecting functionally relevant DNA elements. Thereby, it creates an ESC chromatin architecture that supports ESC specific gene expression programs.

With several ChIP-chip and ChIP-seq data sets published [14], [24], [28], [43], a significant repertoire of cohesin and CTCF binding sites in various cell types is now available. The comparative analysis of CTCF and cohesin binding data published with this manuscript should provide a high confidence list for future studies. Eventually, an integrated analysis also from differentiated cells should allow a refinement of the cohesin binding patterns and may ultimately help to build a roadmap for chromosome architecture during development.

A remaining question is how the transcription factors interact with the cohesin proteins implicated in chromosome conformation. The study of Kagey and colleagues showed interaction of the cohesin loading factor NIPBL with the mediator complex [43], of which the latter phenocopied cohesin in terms of ESC identity. In the study of Tutter and colleagues an interaction of the mediator component Med12 with Nanog was described [46]. Additionally, they found that Med12 and Nanog exhibited similar phenotypes, gene expression profiles and patterns of genome distribution. Another study revealed protein-protein interaction of Oct4 with SMC1a [47]. Our data suggest that Nanog may facilitate the placement of cohesin at its binding sites through an interaction with STAG1 and/or WAPL. Considering the fact that cohesin co-localizes at multiple transcription factor loci, the direct interaction of Oct4 with SMC1a and Nanog with STAG1 and WAPL may support the pluripotency transcription factor mediated placement of cohesin at CTCF-independent binding sites.

Collectively, these data suggest a complex interplay of the different co-localizing protein complexes and strongly indicate that these factors act in concert to maintain embryonic stem cell identity. However, a possible hierarchy of events and functions for the cooperativity of RAD21 and other cohesin subunits with pluripotency transcription factors at these sites needs future investigations. A detailed dissection of interactions between cohesin proteins and pluripotency transcription factors will certainly enhance our understanding of transcriptional regulation in light of higher order chromosome architecture.

## Methods

### Cell culture

Feeder-free mouse R1/E ESC were maintained on gelatin-coated dishes in DMEM (GLUTAMAX high-glucose, Gibco) media supplemented with 15% FBS (Gibco), 0.055 mM β-mercaptoethanol (Gibco), 1× MEM non-essential amino acids (Invitrogen), 5000 u/ml penicillin-streptomycin (Invitrogen) and 16 ng LIF (in house production as previously described [32])/250 ml medium.

### BAC transgenomics

BACs harbouring the genes of interest were obtained from the BACPAC Resource Center (http://bacpac.chori.org; BAC-IDs: RP23-375K15 (RAD21), RP23-236B2 (CTCF) and RP24-230P19 (Nanog)). A LAP cassette [48] was inserted as a C-terminal fusion using recombineering technology [49] (Gene Bridges). Isolated BAC DNA was transfected and selected for stable integration as described [34]. Resistant clones were additionally sorted for GFP-positive cells by FACS. Correct protein size and localization was verified by Western blot and immunofluorescent staining as described previously [34].

### Embryoid body differentiation

RAD21-LAP tagged ESC were differentiated into embryoid bodies for 12 days using the hanging drop method according to the protocol of Linda C. Samuelson and Joseph M. Metzger, Cold Spring Harb Protoc 2006.

### RNA interference

Mouse esiRNAs were produced as described previously [50]. Primer sequences are listed in Table S6. EsiRNA transfection was performed for 48 h using Lipofectamine2000 (Invitrogen) according to the manufacture's instructions with 800 ng esiRNA, 2 ul transfection reagent and 80000 cells per well in 12-well plates.

### Western Blot

Cells were harvested and 10–20 ug of total protein extracts were separated on NuPage 4–12% Bis-tris gels (Invitrogen) and blotted on nitrocellulose membranes (Millipore). Membranes were probed with antibodies against GFP (MPI-CBG antibody and protein production facility, 1∶10000), SMC1 and SMC3 (Bethyl Laboratories, Inc., Cat.No. A300-055A and A300-060A, 1∶5000) and RAD21 (Santa Cruz, sc-56208, 1∶500). GAPDH (Novus Biologicals NB300-221, 1∶50.000) was used as a loading control.

### Alkaline Phosphatase staining

Three days post RNAi, cells were fixed with 4% paraformaldehyde (Sigma), rinsed in PBS and stained using Alkaline Phosphatase Red Microwell substrate (Sigma). Images were acquired with a Canon Power Shot G11 digital camera on the Olympus CKX41 microscope.

Based on the staining intensity, percentages of differentiated, half-differentiated and undifferentiated cells were determined by counting.

### RNA isolation, cDNA synthesis and quantitative real-time PCR

For isolation of total RNA the RNeasy Mini Kit (Qiagen) was used according to the manufacture's protocol including a DNaseI digest. Reverse transcription of 0.5–1 ug RNA was performed using Oligo-dT_12–18_ and Superscript III kit (Invitrogen). Quantitative real-time PCR analysis was carried out using SYBR green master mix (Abgene) and the MxP3000 detection system (Stratagene). Samples were run in triplicate and transcript levels were calculated according to the ΔΔct method with normalization to CyclophilinB. Primer sequences are listed in Table S6.

### Immunoprecipitation and mass spectrometry

Immunoprecipitation reactions were carried out with nuclear extracts from Nanog-LAP ESC as well as wildtype ESC and subjected to shotgun mass spectrometry as previously described [51]. For co-immunoprecipitation analysis, nuclear extracts of Nanog-LAP ESC or wildtype ESC were prepared using nuclear extraction reagents (Pierce) and precipitated using a GFP-antibody (MPI-CBG antibody facility). For the Nanog-LAP ESC line, an additional IgG isotype control antibody (Dianova) was used. Western blot detection was performed using GFP (11814460001, Roche) and STAG1 (sc-54515, SantaCruz) antibodies.

### Microarray analysis

Cells were harvested two days post RNAi and 250 ug of isolated total RNA was labeled with the One-Cycle Target Labeling and Control Reagent Package (Affimetrix) as described in the manufacture's instructions. Probes from 4 biological replicates of RAD21 and Luc RNAi were hybridized on Mouse Genome 430.2.0 arrays (Affimetrix). Image data were analyzed with the GeneChip Operation Software applying Affimetrix default settings. Expression changes were determined by a parametric analysis of variance (ANOVA) after RMA normalization with respect to the probe GC content using Partek Genomics Suite 6.4 (6.09.0129) (Table S4).

Gene Ontology analysis was performed using GenCoDis 2.0 [52], [53].

For calculation of Pearson's correlation factor, published microarray data were used [41], [42].

### ChIP and ChIP-sequencing

RAD21-LAP ESC were crosslinked with 1% formaldehyde for 10 min at room temperature and crosslinking was quenched with 125 mM glycine. Sonicated chromatin with an average size of 500 bp was immunoprecipitated over night using a GFP-antibody (MPI-CBG antibody facility) and control IgG-antibody (Dianova) and immobilized on G-sepharose (GE Healthcare). Specificity of the GFP-antibody has been validated before (Poser et al. 2008, Fig. S1). Eluates were reverse crosslinked followed by RNA and protein digestion. Quantitative real-time PCR analysis was performed with 3 biological replicates of purified DNA. Relative occupancy values were calculated as fold enrichment over control-IgG (or as percent of input recovery occupancy) and normalized to RPL19. Primer sequences are listed in Table S6.

For sequencing, 20 ng of the immunoprecipitated DNA was used to generate the ChIP-seq library according to the manufacture's protocol (Illumina) and sequenced with the Genome Analyzer II (Illumina).

### Peak calling

Peak calling was performed using MACS 1.4beta [54] with the following settings for the RAD21 data: mfold = 8, bw = 150 and a *p*-value threshold of 10^−4^, and with the following settings for the previously generated data [37] : mfold = 8, bw = 150 and a p-value threshold of 10^−5^. Prior peak detection, ChIP-seq fragment coordinates from CTCF, Pou5f1, Nanog, Sox2, Klf4 and Esrrb ChIP-seq data [37] were converted into Mouse NCBI genome build 37 (mm9) using the UCSC *liftOver* tool [55].

### Gene loci identification and genomic distribution

PINKTHING (http://pinkthing.cmbi.ru.nl) software was used for assigning sites to the nearest gene and to determine genomic distribution of identified binding sites. Peak locations were visualized using the University of California Santa Cruz (UCSC) genome browser.

All sequence analyses were conducted based on the Mus musculus NCBI m37 genome assembly (mm9; July 2007) accessed from Ensembl.

### Motif analysis

Sequences from a range of 150 bp around peak summits were extracted with BEDTools package (http://code.google.com/p/bedtools) and used as a basis for further search of occurrences of known transcription factor binding motifs from the JASPAR database [56]. The search was performed using FIMO tool from the MEME suite [57]. Motif occurrences with a *p*-value not exceeding 10^−5^ were considered significant. Gaussian mixture modeling of distances between binding sites and motifs was performed with R package mclust (Fraley & Raftery, 2002).

RAD21 binding peaks that were not overlapping with any of the CTCF peaks, were named CTCF independent RAD21 binding sites (CIB). Peaks that were not bound by CTCF and did not contain the CTCF binding motif in the 150 bp vicinity of the peak summits were considered motif and CTCF binding independent binding sites (MCIB). The binding site and the motif enrichment *p*-values were determined with Fisher's exact test. Enrichments were calculated using all RAD21 binding sites as background.

### Accession number

MIAME compliant microarray and sequencing data from this study have been deposited in the MIAME compliant database GEO with the accession number GSE24030.

## Supporting Information

Figure S1 **Validation of BAC-GFP tagged ESC lines and GFP-antibody for ChIP-sequencing.** (**A**)**:** Immunostaining of RAD21 and CTCF BAC-GFP ESC stably expressing RAD21-LAP or CTCF-LAP confirmed nuclear localization. In the merged picture, GFP (LAP tagged protein) is shown in red, α-Tubulin in green and DNA (DAPI) in blue. Scale bar equals 20 µm. (**B**)**:** Western blot analysis of RAD21 and CTCF BAC- GFP ESC depleted in RAD21 and CTCF, respectively. Two independent esiRNAs and control esiRNA (Luc) were used. GAPDH expression served as protein loading control. (**C**)**:** Western blot analysis of RAD21-LAP immunoprecipitation using GFP-antibody to confirm antibody specificity. Co-immunoprecipitation of the cohesin complex members SMC1a and SMC3 support functionality of the RAD21 BAC transgenic ESCs. (**D**)**:** ChIP of RAD21-LAP cells transfected with control (Luc) and RAD21 esiRNA. Enrichment of selected MCIB sites that appertain to indicated genes was quantified by qPCR and confirmed reduced signals upon RAD21 depletion (n = 2, error bars denote s.d.).
(TIF)

Figure S2 **Identification of RAD21 binding sites and their genome distribution.** (**A**)**:** Table shows the number of identified binding sites using MACS algorithm in dependency of the *p*-value. Numbers are listed for RAD21 and IgG ChIP-samples. Binding sites detected with the *p*-value of 10e-5 were used for further analysis. (**B**)**:** Genome distribution of RAD21 binding sites indicated that the majority of RAD21 sites is located in introns and far distant (>25 kb) from the transcriptional start site. (**C**)**:** *De novo* motif analysis in the 150 bp vicinity of RAD21 peak summits did not reveal a RAD21 specific consensus sequence. Search for known DNA binding motifs in the 150 bp vicinity of peak summits using Jaspar database identified the CTCF motif to be the most abundant. (**D–E**)**:** CTCF ChIP-seq data analysis according to A–C.
(TIF)

Figure S3 **Motifs present in the vicinity of RAD21 binding sites apart from CTCF do not exhibit binding directionality. Motifs in the vicinity of RAD21 apart from CTCF do not exhibit directionality.** The histogram plot of distances between RAD21 binding site and FoxI1 motif sequence in 5′ to 3′ strand direction does not exhibit directionality of RAD21 binding. Black lines are the components of the Gaussian mixture modelling distribution of the distances. Solid lines indicate the most dominant distributions. The red bar indicates RAD21 binding site and the black ellipses indicate expected positions of FoxI1 binding sites.
(TIF)

Figure S4 **Depletion of cohesin strongly changes ESC morphology.** ESC transfected with esiRNAs against RAD21 (2 independent esiRNAs), SMC1a and SMC3 exhibit strong change in morphology 72 h post RNAi compared to non-targeting control (Luc). Depletion of Nanog served as a positive control for ESC differentiation. Scale bars correspond to 100 µm.
(TIF)

Figure S5 **Depletion of SMC1a and SMC3 reflects expression changes upon RAD21 knock-down.** (**A**)**:** Alkaline phosphatase staining of ESCs, which had been transfected with secondary esiRNAs targeting RAD21, SMC1a and SMC3 (72 h post RNAi). Nanog depletion and a non-targeting control (Luc) served as a positive and negative control for ESC differentiation, respectively. Scale bars correspond to 100 µm. (**B+C**)**:** qPCR result of detected expression changes in (**B**) stem cell maintenance genes and (**C**) lineage marker genes upon knock-down of SMC1a and SMC3 versus a Luc control (48 h RNAi, n = 3, error bars denote s.d. and *, **, *** indicate p<0.05, 0.01 and 0.001, respectively).
(TIF)

Figure S6 **Validation of microarray gene expression results.** Diagram shows qPCR based confirmation of up- and downregulation of selected developmental and stem cell maintenance related genes identified in the RAD21 microarray gene expression array (48 h RNAi, n = 3, error bars denote s.d. and *, **, *** indicate p<0.05, 0.01 and 0.001, respectively.
(TIF)

Figure S7 **Re-analysis of SMC1 and SMC3 ChIP-seq data.** Analysis of the SMC1, SMC3 and CTCF DNA binding data. [43] and determination of the overlaps of the individual experiments is shown using MACS 1.4beta (p<10^−5^). Numbers of binding sites are presented for the individual experiments, calculating intersections of each of the two replicates to define CTCF overlapping and independent binding sites. The green dashed line separates cohesin sites that are independent of CTCF binding from sites that are independent of CTCF binding and do not contain the CTCF motif sequence (MCIB, shown in red).
(TIF)

Table S1 RAD21 binding sites in ESCs and EBs.
(XLS)

Table S2 Enrichment analysis of transcription factor binding motifs at RAD21 binding sites in ESCs and EBs.
(XLS)

Table S3 Enrichment analysis of RAD21 co-localizing with ESC transcription factor binding sites in ESCs.
(XLS)

Table S4 Gene expression microarray data of RAD21 depleted ESCs.
(XLS)

Table S5 List of Nanog interacting proteins identified by mass spectrometry.
(XLS)

Table S6 Primer sequences for esiRNA production, qPCR and ChIP.
(XLS)

Table S7 High-confidence cohesin binding sites.
(XLS)
