# Supplementary figures and images for: RAD21 Cooperates with Pluripotency Transcription Factors in the Maintenance of Embryonic Stem Cell Identity
Source: PLoS One. 2011 May 12;6(5):e19470. doi: 10.1371/journal.pone.0019470 (PMC3093395; doi:10.1371/journal.pone.0019470)

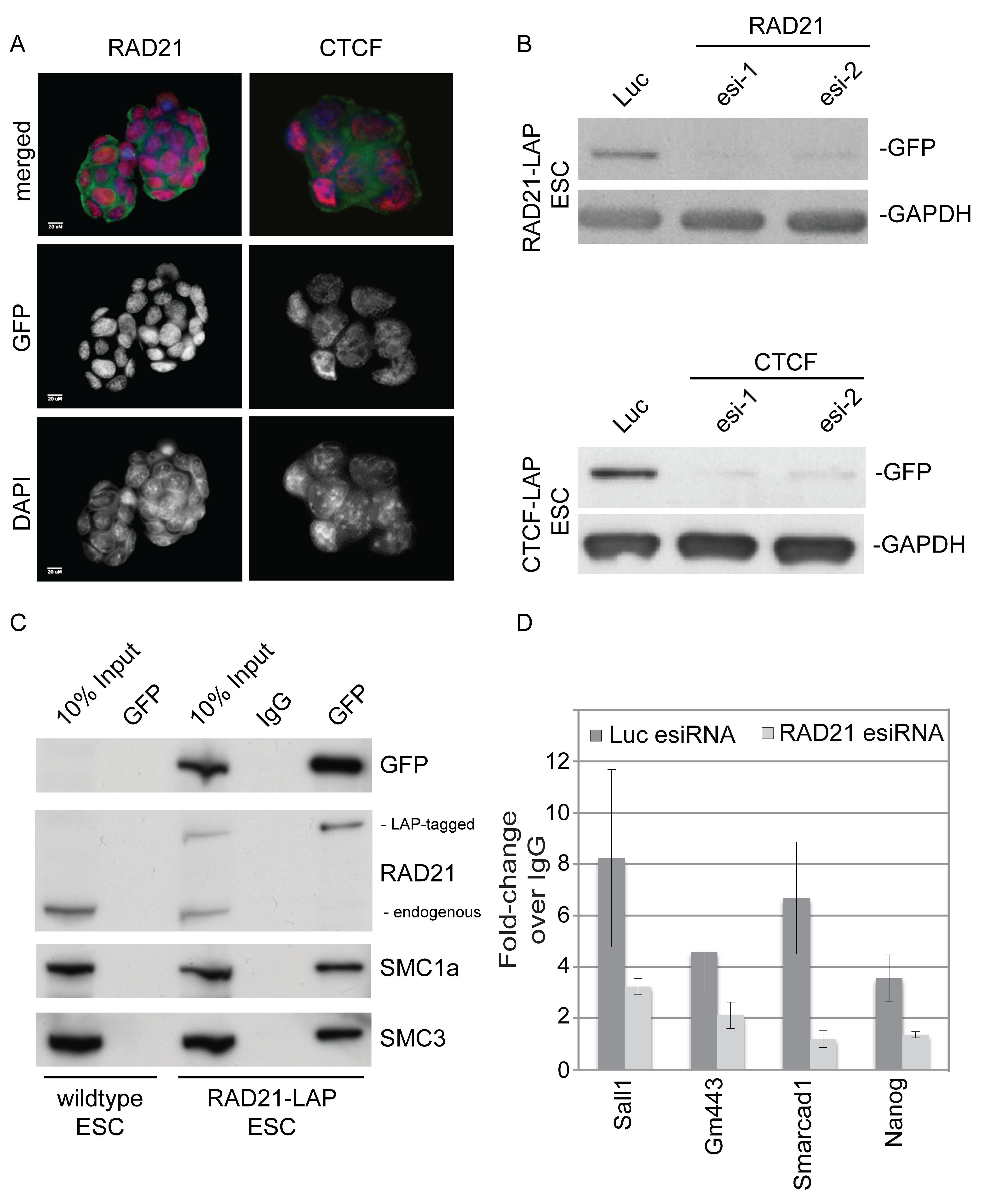

Supplement: Figure S1 — Validation of BAC-GFP tagged ESC lines and GFP-antibody for ChIP-sequencing. (A): Immunostaining of RAD21 and CTCF BAC-GFP ESC stably expressing RAD21-LAP or CTCF-LAP confirmed nuclear localization. In the merged picture, GFP (LAP tagged protein) is shown in red, α-Tubulin in green and DNA (DAPI) in blue. Scale bar equals 20 µm. (B): Western blot analysis of RAD21 and CTCF BAC- GFP ESC depleted in RAD21 and CTCF, respectively. Two independent esiRNAs and control esiRNA (Luc) were used. GAPDH expression served as protein loading control. (C): Western blot analysis of RAD21-LAP immunoprecipitation using GFP-antibody to confirm antibody specificity. Co-immunoprecipitation of the cohesin complex members SMC1a and SMC3 support functionality of the RAD21 BAC transgenic ESCs. (D): ChIP of RAD21-LAP cells transfected with control (Luc) and RAD21 esiRNA. Enrichment of selected MCIB sites that appertain to indicated genes was quantified by qPCR and confirmed reduced signals upon RAD21 depletion (n = 2, error bars denote s.d.). (TIF) [file pone.0019470.s001.tif]

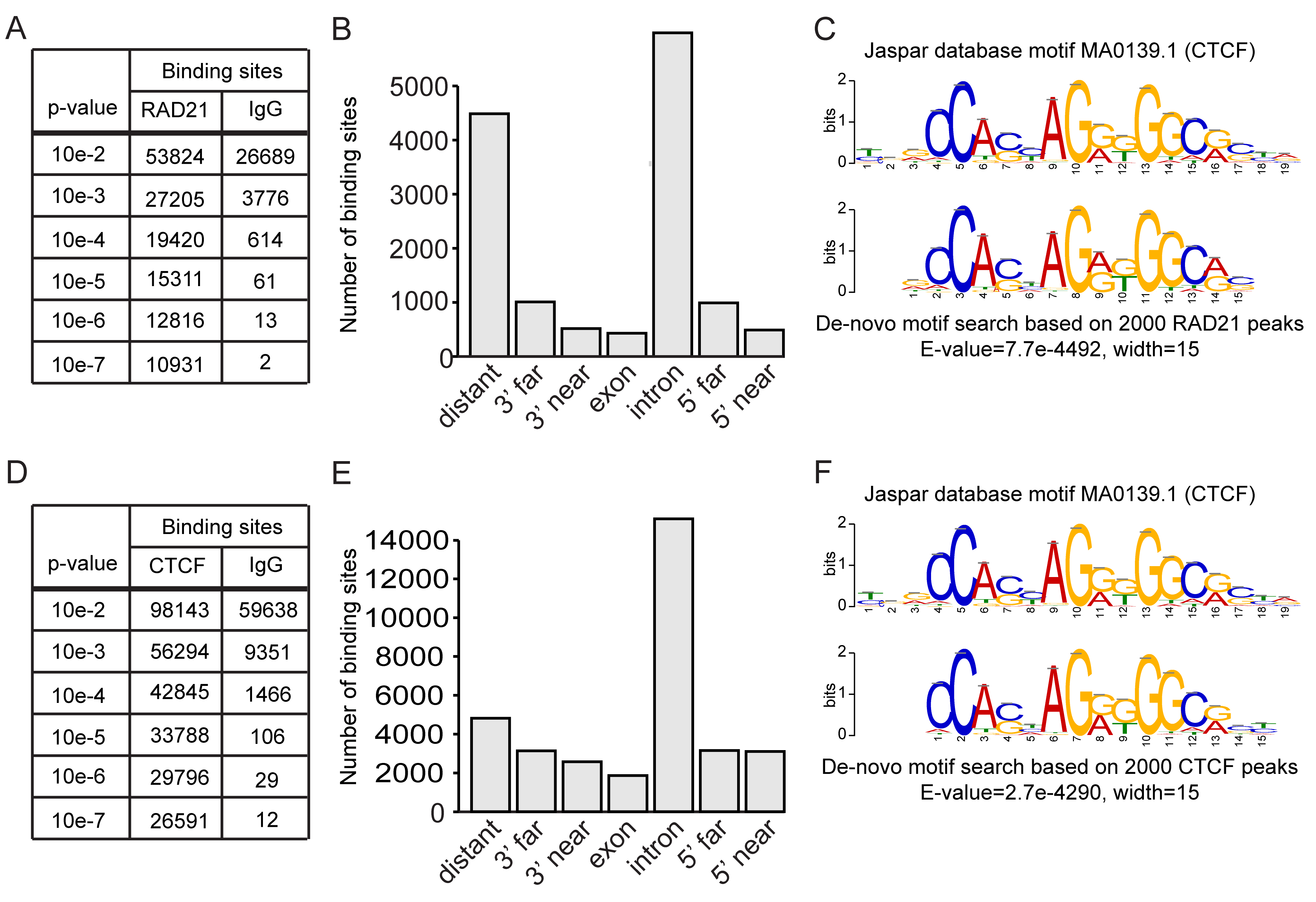

Supplement: Figure S2 — Identification of RAD21 binding sites and their genome distribution. (A): Table shows the number of identified binding sites using MACS algorithm in dependency of the p-value. Numbers are listed for RAD21 and IgG ChIP-samples. Binding sites detected with the p-value of 10e-5 were used for further analysis. (B): Genome distribution of RAD21 binding sites indicated that the majority of RAD21 sites is located in introns and far distant (>25 kb) from the transcriptional start site. (C): De novo motif analysis in the 150 bp vicinity of RAD21 peak summits did not reveal a RAD21 specific consensus sequence. Search for known DNA binding motifs in the 150 bp vicinity of peak summits using Jaspar database identified the CTCF motif to be the most abundant. (D–E): CTCF ChIP-seq data analysis according to A–C. (TIF) [file pone.0019470.s002.tif]

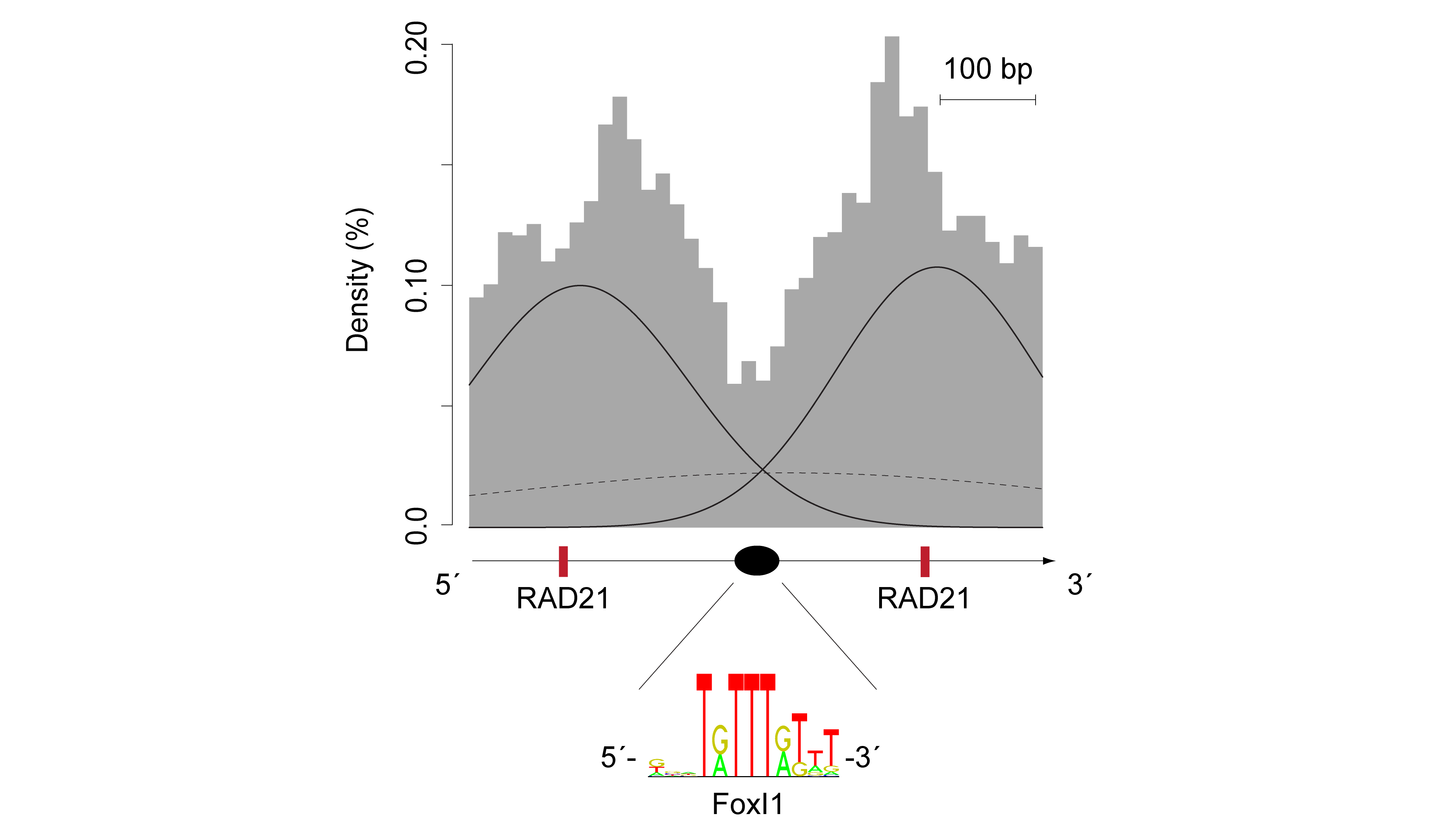

Supplement: Figure S3 — Motifs present in the vicinity of RAD21 binding sites apart from CTCF do not exhibit binding directionality. Motifs in the vicinity of RAD21 apart from CTCF do not exhibit directionality. The histogram plot of distances between RAD21 binding site and FoxI1 motif sequence in 5′ to 3′ strand direction does not exhibit directionality of RAD21 binding. Black lines are the components of the Gaussian mixture modelling distribution of the distances. Solid lines indicate the most dominant distributions. The red bar indicates RAD21 binding site and the black ellipses indicate expected positions of FoxI1 binding sites. (TIF) [file pone.0019470.s003.tif]

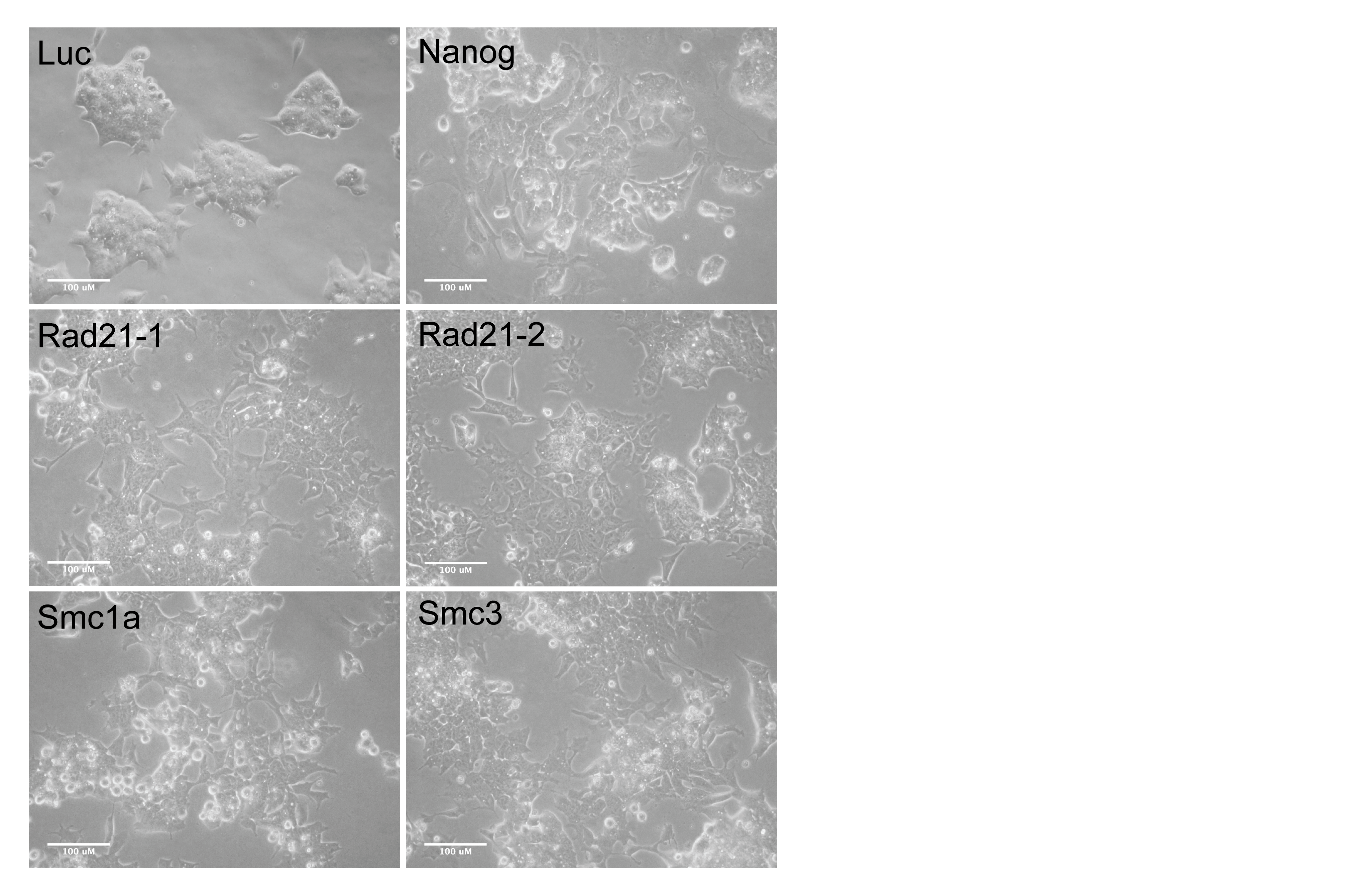

Supplement: Figure S4 — Depletion of cohesin strongly changes ESC morphology. ESC transfected with esiRNAs against RAD21 (2 independent esiRNAs), SMC1a and SMC3 exhibit strong change in morphology 72 h post RNAi compared to non-targeting control (Luc). Depletion of Nanog served as a positive control for ESC differentiation. Scale bars correspond to 100 µm. (TIF) [file pone.0019470.s004.tif]

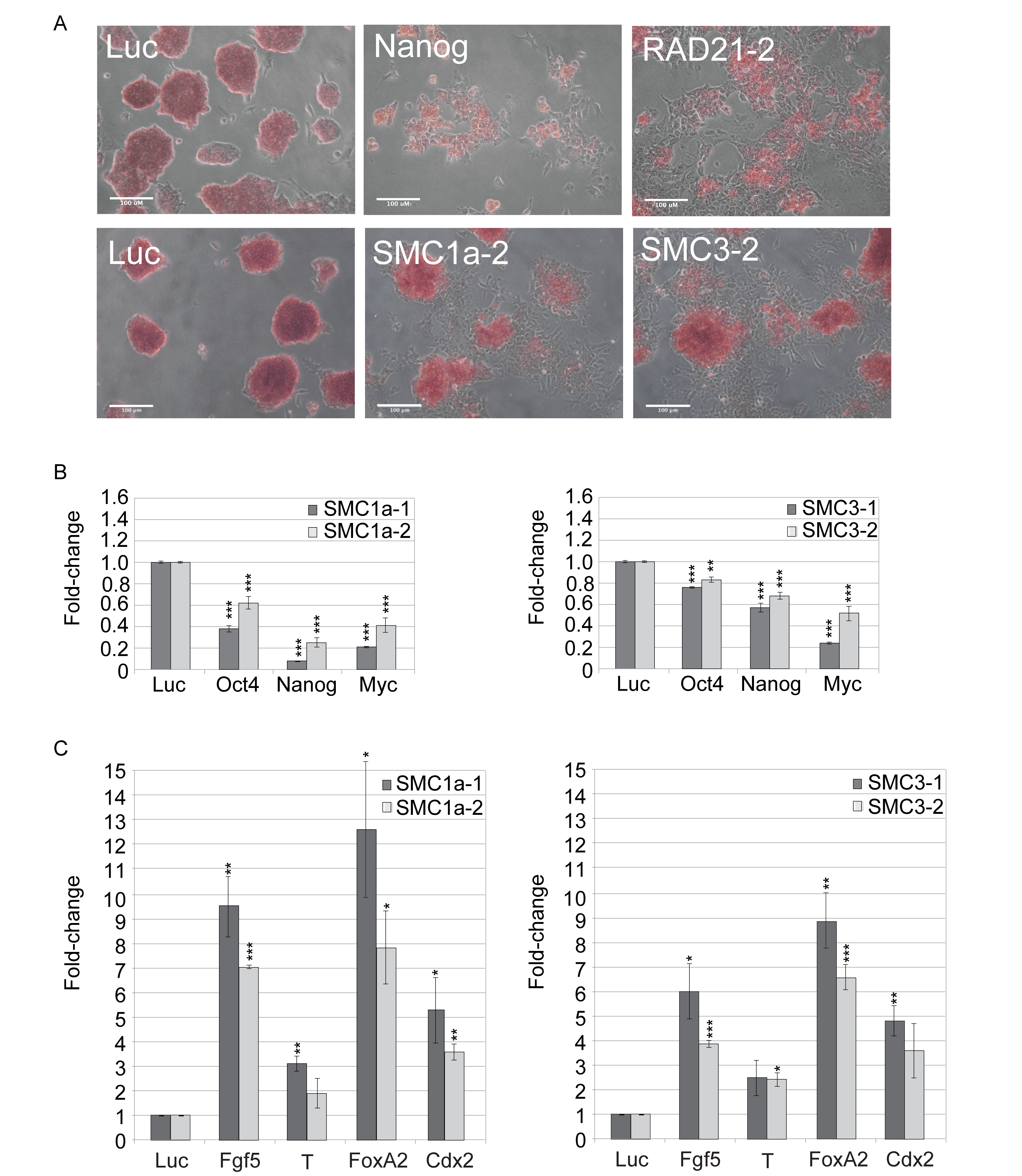

Supplement: Figure S5 — Depletion of SMC1a and SMC3 reflects expression changes upon RAD21 knock-down. (A): Alkaline phosphatase staining of ESCs, which had been transfected with secondary esiRNAs targeting RAD21, SMC1a and SMC3 (72 h post RNAi). Nanog depletion and a non-targeting control (Luc) served as a positive and negative control for ESC differentiation, respectively. Scale bars correspond to 100 µm. (B+C): qPCR result of detected expression changes in (B) stem cell maintenance genes and (C) lineage marker genes upon knock-down of SMC1a and SMC3 versus a Luc control (48 h RNAi, n = 3, error bars denote s.d. and *, **, *** indicate p<0.05, 0.01 and 0.001, respectively). (TIF) [file pone.0019470.s005.tif]

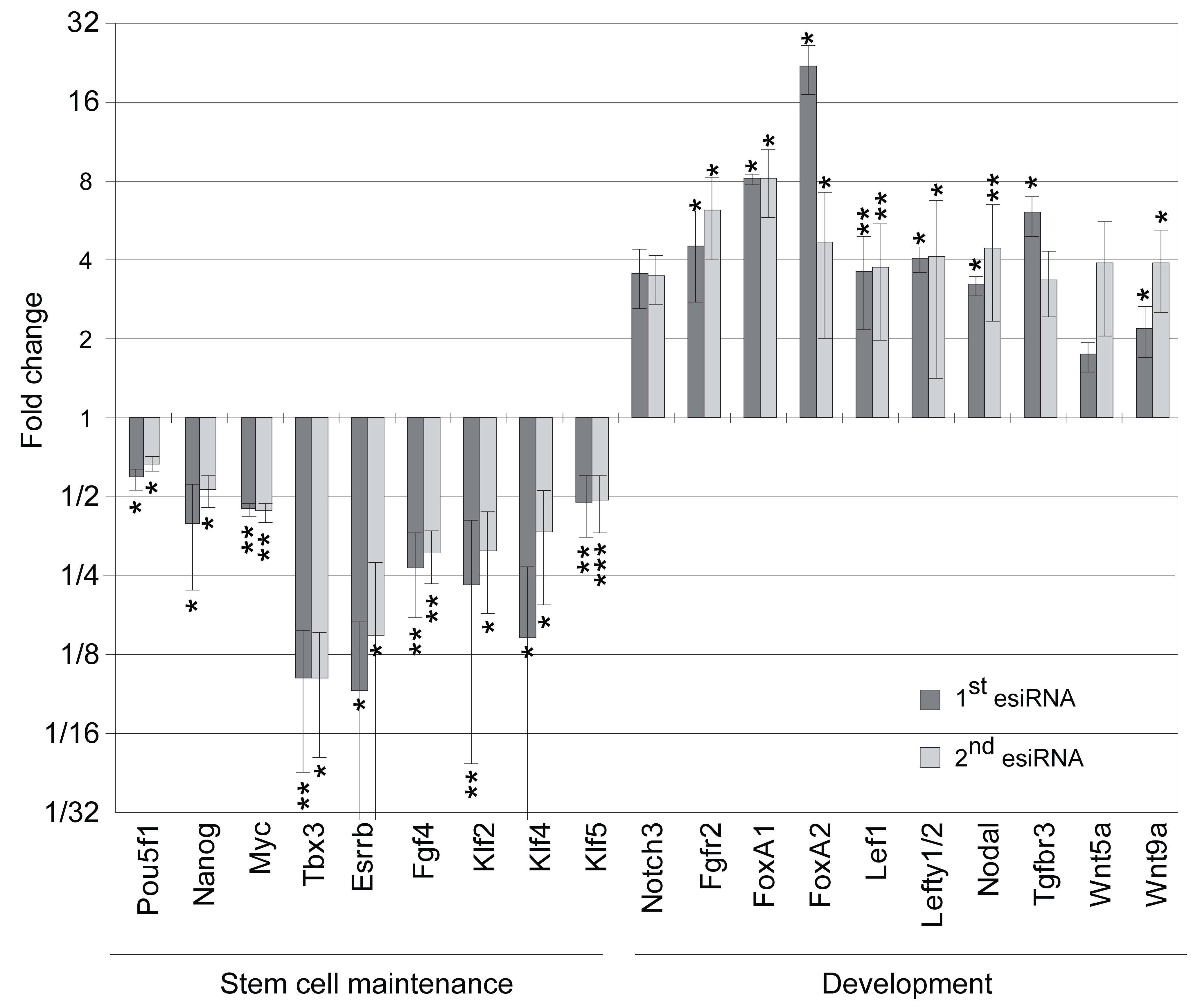

Supplement: Figure S6 — Validation of microarray gene expression results. Diagram shows qPCR based confirmation of up- and downregulation of selected developmental and stem cell maintenance related genes identified in the RAD21 microarray gene expression array (48 h RNAi, n = 3, error bars denote s.d. and *, **, *** indicate p<0.05, 0.01 and 0.001, respectively. (TIF) [file pone.0019470.s006.tif]

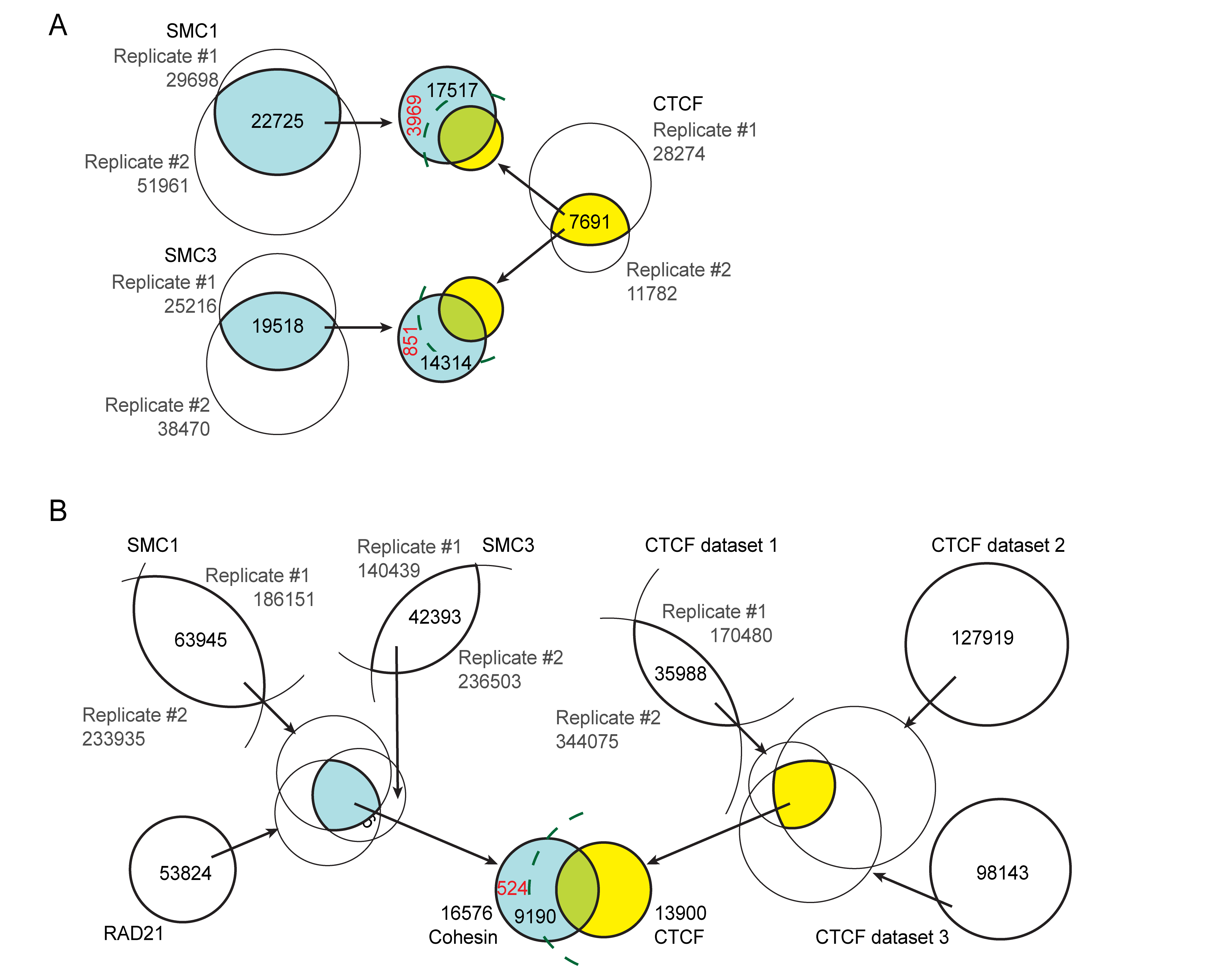

Supplement: Figure S7 — Re-analysis of SMC1 and SMC3 ChIP-seq data. Analysis of the SMC1, SMC3 and CTCF DNA binding data. [43] and determination of the overlaps of the individual experiments is shown using MACS 1.4beta (p<10−5). Numbers of binding sites are presented for the individual experiments, calculating intersections of each of the two replicates to define CTCF overlapping and independent binding sites. The green dashed line separates cohesin sites that are independent of CTCF binding from sites that are independent of CTCF binding and do not contain the CTCF motif sequence (MCIB, shown in red). (TIF) [file pone.0019470.s007.tif]
